# Genetic evidence of the causal relationship between chronic liver diseases and musculoskeletal disorders

**DOI:** 10.1186/s12967-024-04941-1

**Published:** 2024-02-06

**Authors:** Zhengjie Lu, Xuefei Li, Yongjian Qi, Bin Li, Liaobin Chen

**Affiliations:** 1 Division of Joint Surgery and Sports Medicine, Department of Orthopedic Surgery, Zhongnan Hospital of Wuhan University, Wuhan, 430000 China; 2 Department of Pathology, Union Hospital, Tongji Medical College, Huazhong University of Science and Technology, Wuhan, 430022 China; 3 Department of Spine Surgery and Musculoskeletal Tumor, Department of Orthopedic Surgery, Zhongnan Hospital of Wuhan University, Wuhan, 430071 China

**Keywords:** Chronic liver disease, Musculoskeletal disorder, Mendelian randomization, Causal relationship, Primary sclerosing cholangitis, Hepatocellular carcinoma

## Abstract

**Background:**

Chronic liver diseases constitute a major global public health burden, posing a substantial threat to patients’ daily lives and even survival due to the potential development of musculoskeletal disorders. Although the relationship between chronic liver diseases and musculoskeletal disorders has received extensive attention, their causal relationship has not been comprehensively and systematically investigated.

**Methods:**

This study aimed to assess the causal relationships between viral hepatitis, primary biliary cholangitis, primary sclerosing cholangitis (PSC), liver cirrhosis, and hepatocellular carcinoma (HCC) with osteoporosis, osteoarthritis, and sarcopenia through bidirectional Mendelian randomization (MR) research. The traits related to osteoporosis and osteoarthritis included both overall and site-specific phenotypes, and the traits linked to sarcopenia involved indicators of muscle mass and function. Random-effect inverse-variance weighted (IVW), weighted median, MR-Egger, and Causal Analysis Using the Summary Effect Estimates were used to evaluate causal effects, with IVW being the main analysis method. To enhance robustness, sensitivity analyses were performed using Cochran’s Q test, MR-Egger intercept, MR-PRESSO global test, funnel plots, leave-one-out analyses, and latent causal variable model.

**Results:**

The forward MR analysis indicated that PSC can reduce forearm bone mineral density (beta = − 0.0454, 95% CI − 0.0798 to − 0.0110; *P* = 0.0098) and increase the risk of overall osteoarthritis (OR = 1.012, 95% CI 1.002–1.022; *P* = 0.0247), while HCC can decrease grip strength (beta = − 0.0053, 95% CI − 0.008 to − 0.0025; *P* = 0.0002). The reverse MR analysis did not find significant causal effects of musculoskeletal disorders on chronic liver diseases. Additionally, no heterogeneity or pleiotropy was detected.

**Conclusions:**

These findings corroborate the causal effects of PSC on osteoporosis and osteoarthritis, as well as the causal impact of HCC on sarcopenia. Thus, the implementation of comprehensive preventive measures is imperative for PSC and HCC patients to mitigate the risk of musculoskeletal disorders, ultimately improving their quality of life.

**Supplementary Information:**

The online version contains supplementary material available at 10.1186/s12967-024-04941-1.

## Introduction

Chronic liver disease is a condition marked by liver cell damage and aberrant liver function [1]. This ailment is responsible for roughly two million global fatalities annually, constituting a major burden on global public health [2, 3]. Conditions such as viral hepatitis and nonalcoholic fatty liver disease (NAFLD), along with autoimmune liver diseases like primary biliary cholangitis (PBC) and primary sclerosing cholangitis (PSC), have the potential to evolve into liver cirrhosis and even hepatocellular carcinoma (HCC), resulting in grave adverse outcomes [4, 5].

The constellation of systemic symptoms that manifest during the progression of chronic liver diseases is the main culprits behind patients’ functional impairments and diminished quality of life, rendering it a significant clinical concern [6]. Musculoskeletal disorders which may occur alongside chronic liver diseases are of particular concern, given their association with severe adverse outcomes such as mortality, attracting widespread attention [7]. Epidemiological investigations have pointed to an increased risk of osteoporosis [8–12] and sarcopenia [11–13] among patients with chronic liver diseases. For example, clinical studies have found that the prevalence of osteoporosis in patients with liver cirrhosis, PSC, and PBC is 34.5%, 15%, and 28.2%, respectively [9, 11, 12], while the prevalence of sarcopenia in patients with HCC, liver cirrhosis, and PBC is as high as 28.0%, 28.2%, and 23.1%, respectively [11–13]. However, some epidemiological studies have failed to establish a significant link between chronic liver diseases and musculoskeletal disorders, such as osteoarthritis and sarcopenia [14, 15]. Consequently, the exact relationship between chronic liver diseases and musculoskeletal disorders remains inconclusive, which hinders the improvement of long-term quality of life for patients with chronic liver diseases. Recognizing the challenges posed by confounding factors and reverse causality in traditional observational studies, coupled with the ethical and cost-related obstacles faced by randomized controlled trials (RCTs), there exists an urgent imperative to employ alternative strategies to elucidate the causal association between chronic liver diseases and musculoskeletal disorders.

Chronic liver diseases and musculoskeletal disorders exhibit a certain degree of involvement of genetic heritability in disease development. For instance, the heritability of PBC and PSC is estimated at 37.2% and 14.8% respectively [16, 17], while the heritability of indicators related to osteoporosis, osteoarthritis, and sarcopenia is 25.9%, 11.0%, and 4.4%, respectively [18–20]. Genome-wide association studies (GWAS) have identified numerous genetic variations for chronic liver diseases and musculoskeletal disorders in large populations, greatly facilitating Mendelian randomization (MR) research on them. MR analysis is a potent epidemiological tool widely employed to establish causal connections between risk factors and diseases [21]. Utilizing genetic variants randomly assigned at birth as instrumental variables (IVs), MR analysis can substantially mitigate bias arising from confounding factors and reverse causality [22]. Additionally, MR analysis offers a means to circumvent the high costs, time-consuming nature, and ethical issues associated with RCTs. Therefore, MR analysis is an effective strategy for probing the causal link between chronic liver diseases and musculoskeletal disorders. Prior MR studies have already identified a causal relationship between NAFLD and osteoporosis [23] but found no significant correlation with sarcopenia [24]. However, these studies have not comprehensively revealed the causal relationship between chronic liver diseases and musculoskeletal disorders.

This study utilized large-scale GWAS data and employed a bidirectional two-sample MR analysis to investigate the causal relationships between a spectrum of chronic liver diseases and diverse musculoskeletal disorders, which is conducive to facilitating comprehensive treatment for patients with chronic liver diseases and enhancing their quality of life.

## Methods

### Study design

**Fig. 1:**
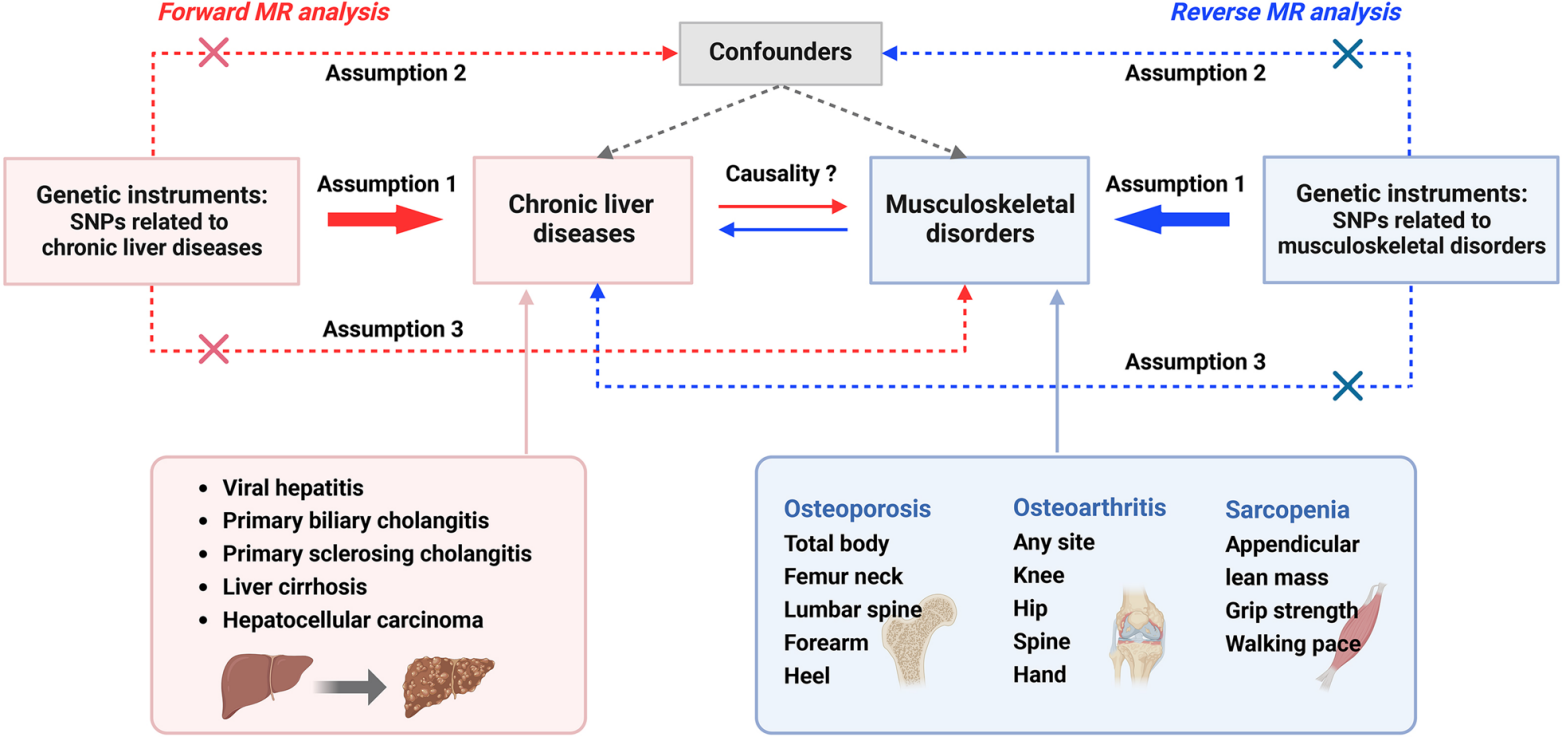
Study design overview. MR, Mendelian randomization; SNPs, single nucleotide polymorphisms

This research is a bidirectional two-sample MR study. MR analysis was conducted in two directions (Fig. 1): (i) using chronic liver diseases as the “exposure” to explore their causal effects on musculoskeletal disorders; (ii) using musculoskeletal disorders as the “exposure” to evaluate their causal impact on chronic liver diseases. MR analysis relies on three fundamental assumptions (Fig. 1): (i) genetic variants exhibit associations with the risk factor; (ii) genetic variants remain independent of confounding factors; (iii) genetic variants exert their influence on the outcome exclusively through the risk factor. This study was structured around three phases: the selection of IVs, MR analysis, and sensitivity analysis.

### Data sources for chronic liver diseases and musculoskeletal disorders

The chronic liver diseases considered in this study encompassed viral hepatitis, PBC, PSC, liver cirrhosis, and HCC. GWAS data pertinent to viral hepatitis, liver cirrhosis, and HCC were sourced from the FinnGen Study (R9 version). FinnGen Study is a nationwide Finnish GWAS meta-analysis amalgamating imputed genotype data generated from newly collected and legacy samples from Finnish biobanks and digital health record data from Finnish health registries [25]. The identification of cases for viral hepatitis followed the classification of ICD-10 codes B15-B19, while controls refer to individuals other than cases. Cases for liver cirrhosis were identified by the ICD-10 code K74.6, and the controls were selected from the population without any broadly defined cirrhosis. Cases for HCC were identified using the ICD-10 code C22.0, and controls excluded individuals with any type of cancer. The detailed definitions are provided in Additional file 1**: **Table S1. The IVs associated with PBC were extracted from the largest international genome-wide meta-analysis of PBC to date, which included five European cohorts [26]. For PSC, the single nucleotide polymorphisms (SNPs) were obtained from the most extensive PSC GWAS conducted by the International PSC Study Group [17]. Table 1 provides detailed information about the sources of the GWAS data mentioned above.

**Table 1:** Characteristics of the genome-wide association study summary data

Phenotype	Sample size	Ethnicity	Consortium/Cohort	Year of publication	PMID
Chronic liver diseases					
Viral hepatitis	377277 (2143 cases/375134 controls)	European	FinnGen R9	2022	36653562
PBC	24510 (8021 cases/16489 controls)	European	NA	2021	34033851
PSC	14890 (2871 cases/12019 controls)	European	IPSCSG	2017	27992413
Liver cirrhosis	374449 (1142 cases/373307 controls)	European	FinnGen R9	2022	36653562
HCC	287590 (453 cases/287137 controls)	European	FinnGen R9	2022	36653562
Musculoskeletal disorders					
Osteoporosis					
TB-BMD	56284	European	GEFOS	2018	29304378
FN-BMD	32735	European	GEFOS	2015	26367794
LS-BMD	28489	European	GEFOS	2015	26367794
FA-BMD	8143	European	GEFOS	2015	26367794
eBMD	426824	European	UK Biobank	2019	30598549
Osteoarthritis					
ALL OA	826690 (177517 cases/649173 controls)	European (~ 98%)	GO Consortium	2021	34822786
Knee OA	396054 (62497 cases/333557 controls)	European (~ 98%)	GO Consortium	2021	34822786
Hip OA	353388 (36445 cases/316943 controls)	European (~ 98%)	GO Consortium	2021	34822786
Spine OA	333950 (28372 cases/305578 controls)	European (~ 98%)	GO Consortium	2021	34822786
Hand OA	303782 (20901 cases/282881 controls)	European (~ 98%)	GO Consortium	2021	34822786
Sarcopenia					
ALM	450243	European	UK Biobank	2020	33097823
Grip strength	461089	European	MRC-IEU	2018	NA
Walking pace	459915	European	MRC-IEU	2018	NA

PBC: primary biliary cholangitis; PSC: primary sclerosing cholangitis; IPSCSG: International PSC Study Group; HCC: hepatocellular carcinoma; TB-BMD: total body bone mineral density; GEFOS: Genetic Factors for osteoporosis Consortium; FN-BMD: femur neck bone mineral density; LS-BMD: lumbar spine bone mineral density; FA-BMD: forearm bone mineral density; eBMD: heel bone mineral density; ALL OA: any site osteoarthritis; GO: Genetics of Osteoarthritis; OA: osteoarthritis; ALM: appendicular lean mass; MRC-IEU: Medical Research Council Integrative Epidemiology Unit

This study investigated three musculoskeletal disorders: osteoporosis, osteoarthritis, and sarcopenia. Osteoporosis is characterized by a reduction in bone mineral density (BMD), which is the most clinically relevant risk factor for diagnosing osteoporosis [27, 28]. GWAS data of total body BMD (TB-BMD), femur neck BMD (FN-BMD), lumbar spine BMD (LS-BMD), and forearm BMD (FA-BMD) were procured from the meta-analysis conducted by the Genetic Factors for osteoporosis Consortium [27, 29], while summary statistics for heel BMD (eBMD) were sourced from the discovery GWAS of UK Biobank [30]. Summary data for osteoarthritis, both overall and specific to the knee, hip, spine, and hand, was derived from a meta-analysis of 13 independent cohorts covering 826690 individuals [31]. Appendicular lean mass (ALM), grip strength, and walking pace are effective predictors of sarcopenia [32]. GWAS data about ALM were obtained from UK Biobank [33], and data for grip strength and walking pace were acquired from Medical Research Council Integrative Epidemiology Unit [34]. Detailed information regarding the sources of the GWAS data mentioned above can be found in Table 1.

Download links to the above GWAS data are shown in Additional file 1: Table S2.

### Selection of IVs

**Fig. 2:**
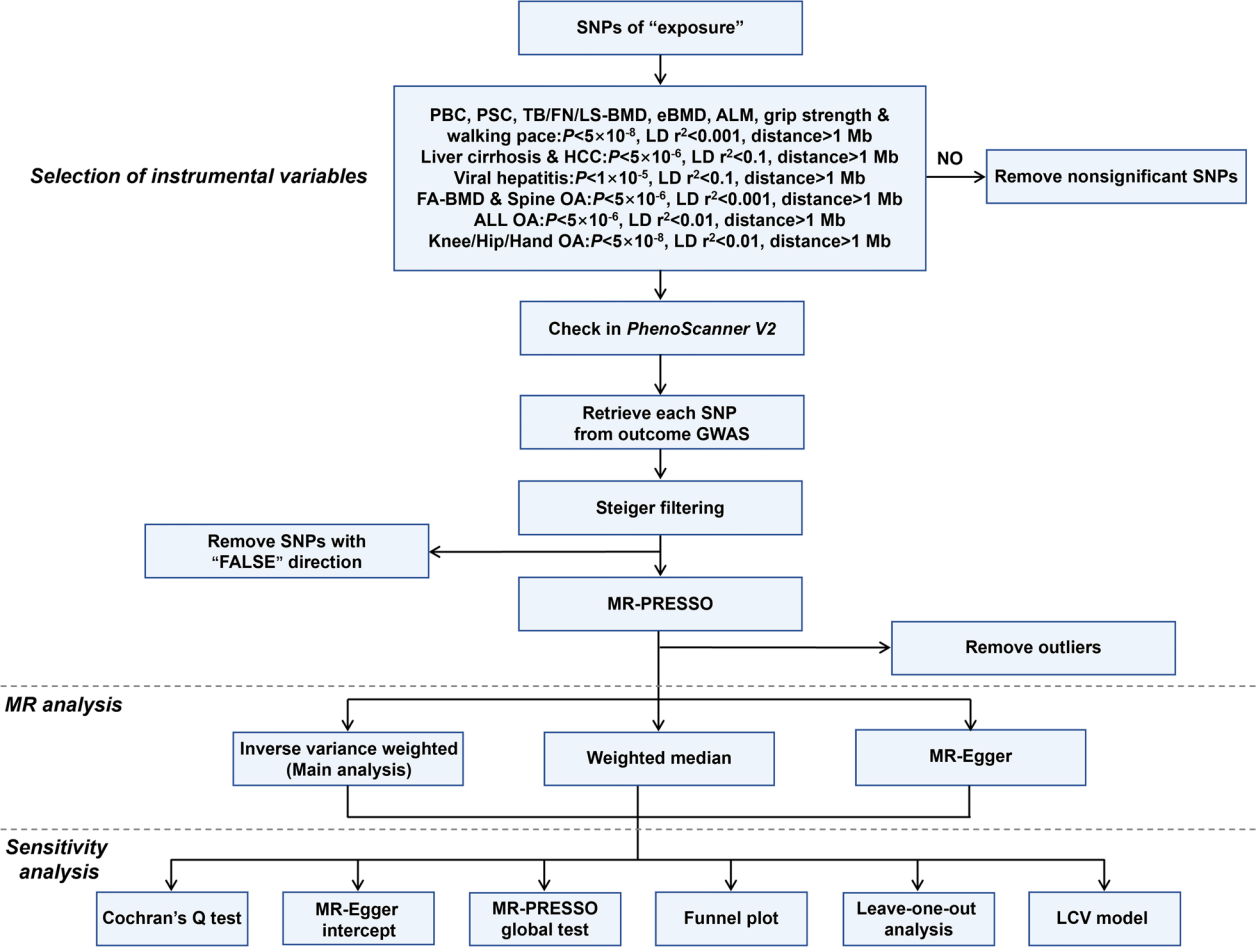
Research process of this Mendelian randomization study. SNPs: single nucleotide polymorphisms; PBC: primary biliary cholangitis; PSC: primary sclerosing cholangitis; TB-BMD: total body bone mineral density; FN-BMD: femur neck bone mineral density; LS-BMD: lumbar spine bone mineral density; eBMD: heel bone mineral density; ALM: appendicular lean mass; LD: linkage disequilibrium; HCC: hepatocellular carcinoma; FA-BMD: forearm bone mineral density; OA: osteoarthritis; ALL OA: any site osteoarthritis; GWAS: genome-wide association study; MR-PRESSO: MR Pleiotropy RESidual Sum and Outlier; MR: Mendelian randomization; LCV: latent causal variable

The selection of valid IVs was carried out through a multi-step process, guided by the three fundamental assumptions (Fig. 2). First, SNPs linked to the exposures were screened based on genome-wide significance threshold *P* value, linkage disequilibrium (LD) r^2^, and distance threshold: *P* value is at least less than 1 × 10^–5^; LD r^2^ is at least less than 0.1; the distance is greater than 1 Mb. Different criteria were set for different exposures to ensure a sufficient number of SNPs [22]. Subsequently, to minimize the influence of confounding factors, SNPs associated with risk factors of the outcomes were excluded by searching in PhenoScanner V2 [35]. Furthermore, the selected exposure-related SNPs were retrieved from the GWAS data of the outcomes. We applied Steiger filtering to test the direction of causality for each SNP on exposure and outcome and removed SNPs which explained more variation in the outcome than in the exposure (“FALSE” direction) [36]. Finally, MR Pleiotropy RESidual Sum and Outlier (MR-PRESSO) was performed to eliminate potential outlier SNPs [37]. In addition, the strength of IVs was assessed by calculating R^2^ and the *F* statistic. An *F* statistic exceeding 10 was considered unlikely to be influenced by weak instrument bias [38].

### MR analysis

To assess the causal relationships between multiple chronic liver diseases and osteoporosis, osteoarthritis, and sarcopenia, two-sample MR analyses were executed. Three analysis methods were employed, including random-effect inverse-variance weighted (IVW), weighted median, and MR-Egger (Fig. 2). The IVW approach assumes the validity of all genetic variants, complying with the three fundamental assumptions, which is considered the most robust MR method [39]. Thus, IVW was used as the primary analysis method in this study. However, this method is prone to bias when horizontal pleiotropy is present across the majority of IVs [39]. The weighted median approach assumes that at least 50% of genetic variants are valid and is suitable for situations where most IVs do not exhibit horizontal pleiotropy [40]. In contrast, MR-Egger assumes that more than 50% of genetic variants are invalid and is better suited for scenarios where horizontal pleiotropy is prevalent among most IVs [41]. Both weighted median and MR-Egger can furnish more dependable estimates in a broader array of scenarios, offering valuable supplements to the IVW analysis. In addition, the Causal Analysis Using the Summary Effect Estimates (CAUSE) method was used to further confirm significant causal relationships. This method estimates the difference in the expected log pointwise posterior density (ΔELPD) to compare the fit of the sharing model and the causal model. CAUSE method utilizes full genome-wide summary results rather than just the genome-wide significant loci, which can correct the bias due to correlated and uncorrelated horizontal pleiotropy and sample overlap [42].

### Sensitivity analysis

This study incorporated a range of sensitivity analysis methods to evaluate the robustness of the results (Fig. 2). Cochran’s Q test for IVW and MR-Egger was utilized to detect any heterogeneity. The intercept of MR-Egger was used to assess the presence of horizontal pleiotropy, thereby ensuring that IVs only affect the outcomes through their corresponding exposures. MR-PRESSO was also used to detect horizontal pleiotropy [43]. The funnel plot was employed as a visual tool to evaluate directional pleiotropy. Additionally, to determine whether the causal relationship was driven by a single SNP, a leave-one-out analysis was conducted by discarding each SNP in turn and re-performing the IVW analysis. Furthermore, the latent causal variable (LCV) model was performed to estimate the genetic causality proportion (GCP) of genetically correlated traits. GCP equal to 1 indicates full causality, while values near 0 indicate partial causality [44].

### Statistical analysis

MR estimates were presented as beta coefficients, odds ratios (OR), and their corresponding 95% confidence intervals (CI). A Bonferroni-corrected significance level of *P* < 0.05/(13 × 5) (*P* < 7.69 × 10^–4^) was applied to identify significant causal relationships, while *P* values ranging from 7.69 × 10^–4^ and 0.05 signified suggestive causal associations [45, 46]. To enhance the reliability of the conclusions, a causal effect of the “exposure” on the “outcome” was considered valid only when all three MR methods produced results in the same direction [37, 47]. In the context of the CAUSE method, statistical significance was determined by a threshold of *P* < 0.05. All analyses were conducted using the TwoSampleMR package (version 0.5.7) and the CAUSE package (version 1.2.0.0335) in R (version 4.3.0).

## Results

### Selection of IVs

Following a series of rigorous screening steps, a total of 5 to 33 IVs linked to the five chronic liver diseases were identified (Additional file 1: Table S3). The phenotypic variance explained (PVE) by these IVs for their respective chronic liver diseases spanned from 0.06% to 15.77%, with average and median PVE being 3.65% and 0.19%, respectively (Additional file 1: Table S3). Furthermore, the *F* statistic ranged from 20.86 to 172.25, with an average of 53.36 and a median of 35.14 (Additional file 1: Table S3). Importantly, the *F* statistic for any single IV was greater than 10 (Additional file 1: Tables S4–6), indicating that they were not affected by weak instrument bias.

In addition, a total of 3 to 574 IVs were identified for phenotypes related to the three musculoskeletal disorders (Additional file 1: Table S7). The PVE by these IVs for their corresponding musculoskeletal disorders ranged from 0.02% to 17.01%, with average and median PVE being 2.78% and 0.98% **(**Additional file 1: Table S7). Moreover, the *F* statistic spanned from 23.45 to 168.98, with an average of 58.46 and a median of 47.10 **(**Additional file 1: Table S7). Notably, all individual IVs had *F* statistics exceeding 10 **(**Additional file 1: Tables S8–10), indicating no weak instrument bias.

For comprehensive information on all IVs utilized in the forward and reverse MR analysis, please refer to Additional file 1: Tables S4–6 and Tables S8–10. The SNPs with “FALSE” direction excluded by Steiger filtering can be found in Additional file 1: Table S11. The outlier SNPs detected by MR-PRESSO were shown in Additional file 1: Table S12.

### Causal effects of chronic liver diseases on musculoskeletal disorders

**Fig. 3:**
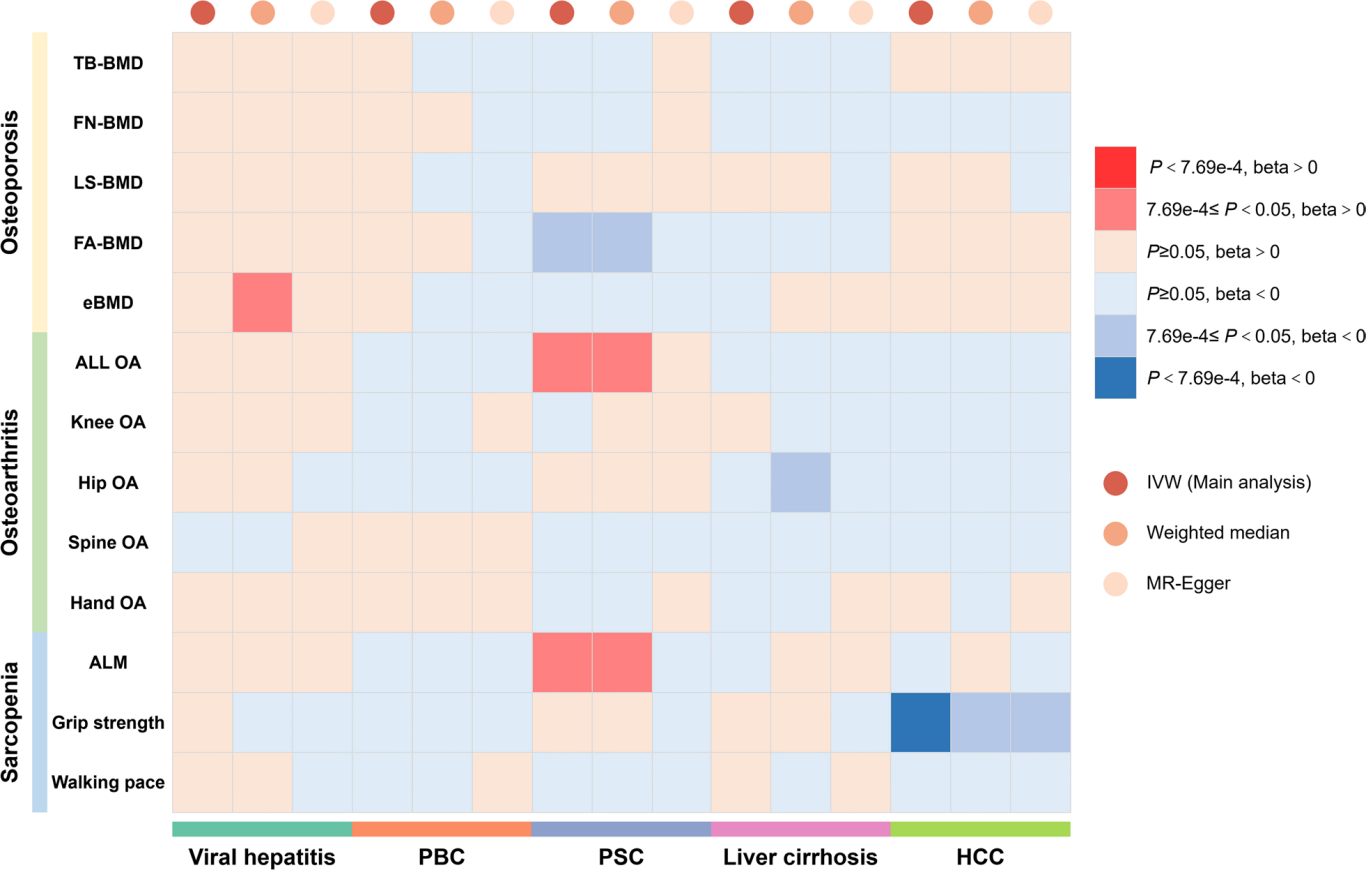
Causal effects of five chronic liver diseases on 13 phenotypes of musculoskeletal disorders. TB-BMD: total body bone mineral density; FN-BMD: femur neck bone mineral density; LS-BMD: lumbar spine bone mineral density; FA-BMD: forearm bone mineral density; eBMD: heel bone mineral density; ALL OA: any site osteoarthritis; OA: osteoarthritis; ALM: appendicular lean mass; PBC: primary biliary cholangitis; PSC: primary sclerosing cholangitis; HCC: hepatocellular carcinoma; IVW: inverse-variance weighted

This study analyzed the causal impact of five chronic liver diseases on 13 phenotypes related to osteoporosis, osteoarthritis, and sarcopenia, using three MR analysis methods. Figure 3 provides a comprehensive summary of this array of causal associations.

#### Causal effects of chronic liver diseases on osteoporosis

**Fig. 4:**
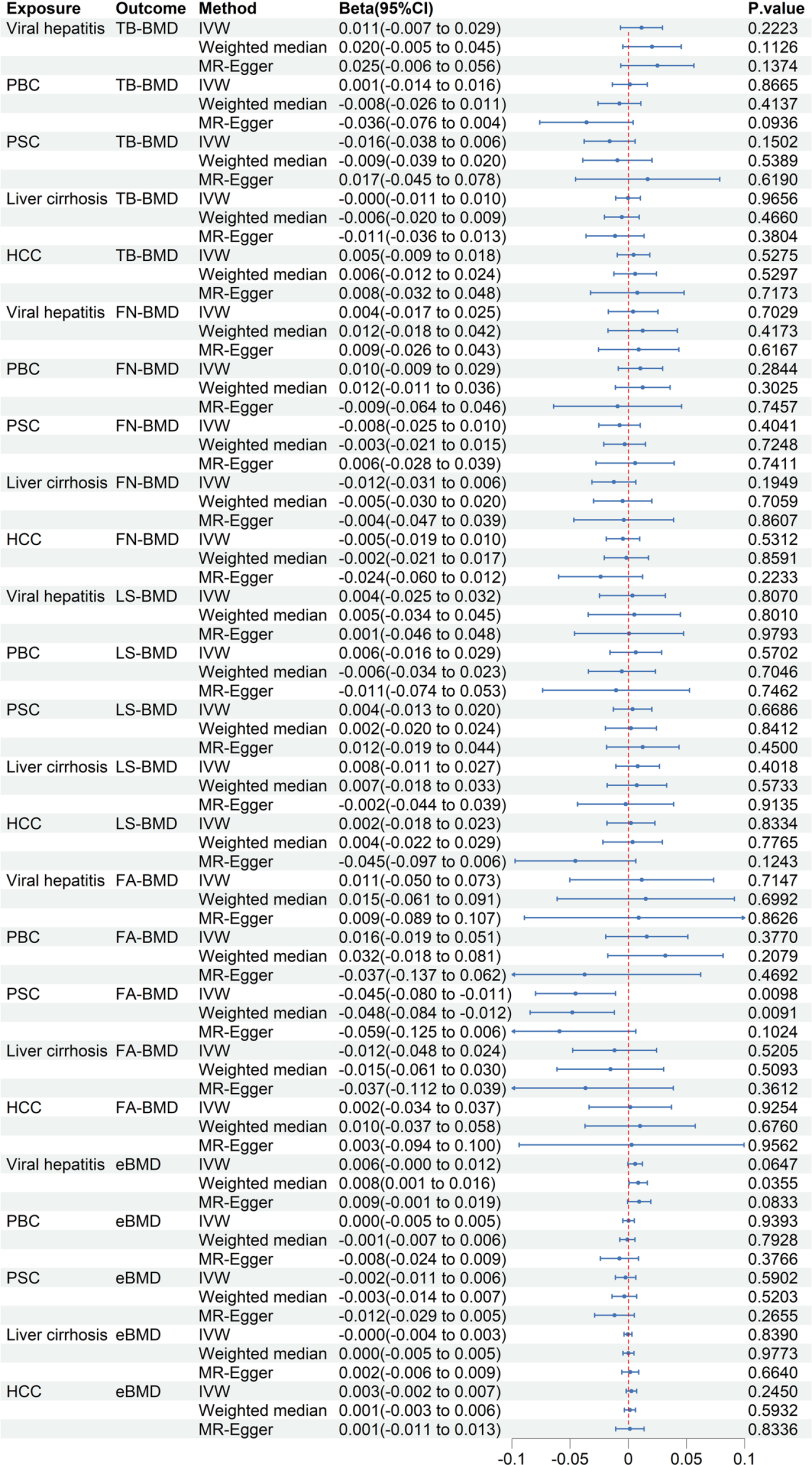
Causal effects of chronic liver diseases on osteoporosis. CI: confidence intervals; TB-BMD: total body bone mineral density; IVW: inverse-variance weighted; PBC: primary biliary cholangitis; PSC: primary sclerosing cholangitis; HCC: hepatocellular carcinoma; FN-BMD: femur neck bone mineral density; LS-BMD: lumbar spine bone mineral density; FA-BMD: forearm bone mineral density; eBMD: heel bone mineral density

For BMD, the IVW analysis showed a suggestive negative association between PSC and FA-BMD (beta = − 0.0454, 95% CI − 0.0798 to − 0.0110; *P* = 0.0098) (Fig. 4). The weighted median analysis also observed a similar negative correlation between PSC and FA-BMD (beta = − 0.0482, 95% CI − 0.0844 to − 0.0120; *P* = 0.0091) (Fig. 4). Meanwhile, MR-Egger analysis yielded results in the same direction but did not reach statistical significance (beta = − 0.0592, 95% CI − 0.1247 to 0.0064; *P* = 0.1024) (Fig. 4). The ΔELPD obtained from the CAUSE method was negative (ΔELPD = − 34.297), indicating that the causal model trended towards a better fit than the sharing model (Additional file 1: Table S13). Meanwhile, the CAUSE method further demonstrated that PSC can reduce FA-BMD (gamma = − 0.18, *P* = 1.5 × 10^–7^) (Additional file 1: Table S13). Additionally, the weighted median analysis indicated a positive causal effect of viral hepatitis on eBMD (beta = 0.0084, 95% CI 0.0006–0.0163; *P* = 0.0355), but the results from the IVW and MR-Egger approaches did not yield statistically significant findings (*P* > 0.05) (Fig. 4). Moreover, no causal effects of viral hepatitis, liver cirrhosis, and HCC on BMD were observed (*P* > 0.05) (Fig. 4).

#### Causal effects of chronic liver diseases on osteoarthritis

**Fig. 5:**
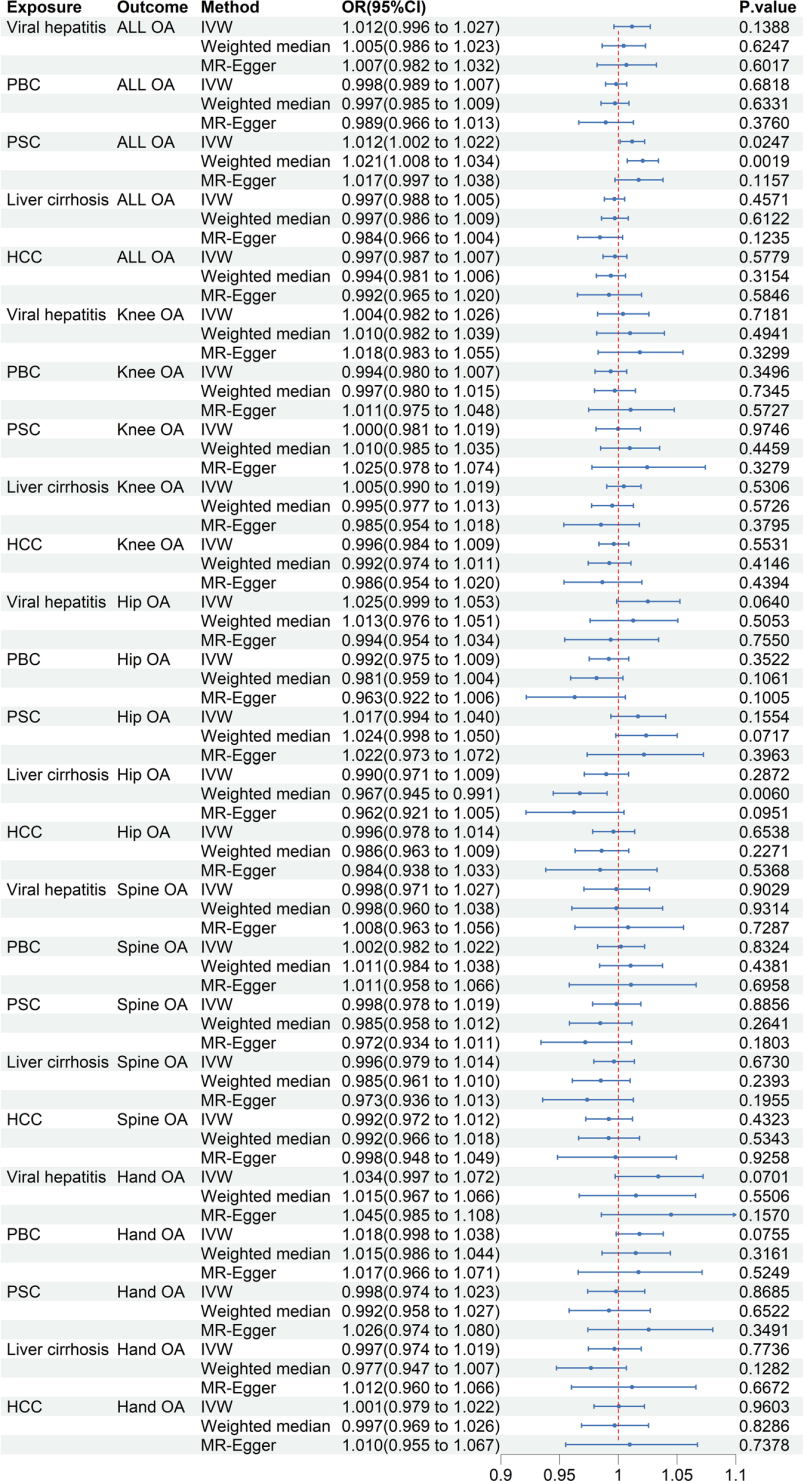
Causal effects of chronic liver diseases on osteoarthritis. OR: odds ratios; CI: confidence intervals; ALL OA: any site osteoarthritis; IVW: inverse-variance weighted; PBC: primary biliary cholangitis; PSC: primary sclerosing cholangitis; HCC: hepatocellular carcinoma; OA: osteoarthritis

Concerning osteoarthritis, the MR analyses showed that PSC can increase the risk of overall osteoarthritis at a suggestive significant level, as indicated by IVW (OR = 1.012, 95% CI 1.002–1.022; *P* = 0.0247) and weighted median (OR = 1.021, 95% CI 1.008–1.034; *P* = 0.0019) (Fig. 5). Although MR-Egger analysis did not yield statistically significant results (OR = 1.017, 95% CI 0.997–1.038; *P* = 0.1157), the consistent direction of results across all three MR methods revealed a positive causal impact of PSC on overall osteoarthritis (Fig. 5). Meanwhile, the CAUSE method provided additional evidence supporting this causal link (ΔELPD = -32.694, gamma = 0.08, *P* = 3.6 × 10^–9^) (Additional file 1: Table S13). Additionally, the weighted median analysis found that liver cirrhosis could decrease the risk of hip osteoarthritis (OR = 0.9673, 95% CI 0.9446–0.9905; *P* = 0.0060), but the results from the IVW and MR-Egger approaches did not reach statistical significance (*P* > 0.05) (Fig. 5). Furthermore, no causal associations were observed between viral hepatitis, PBC, and HCC with either overall or site-specific osteoarthritis (*P* > 0.05) (Fig. 5).

#### Causal effects of chronic liver diseases on sarcopenia

**Fig. 6:**
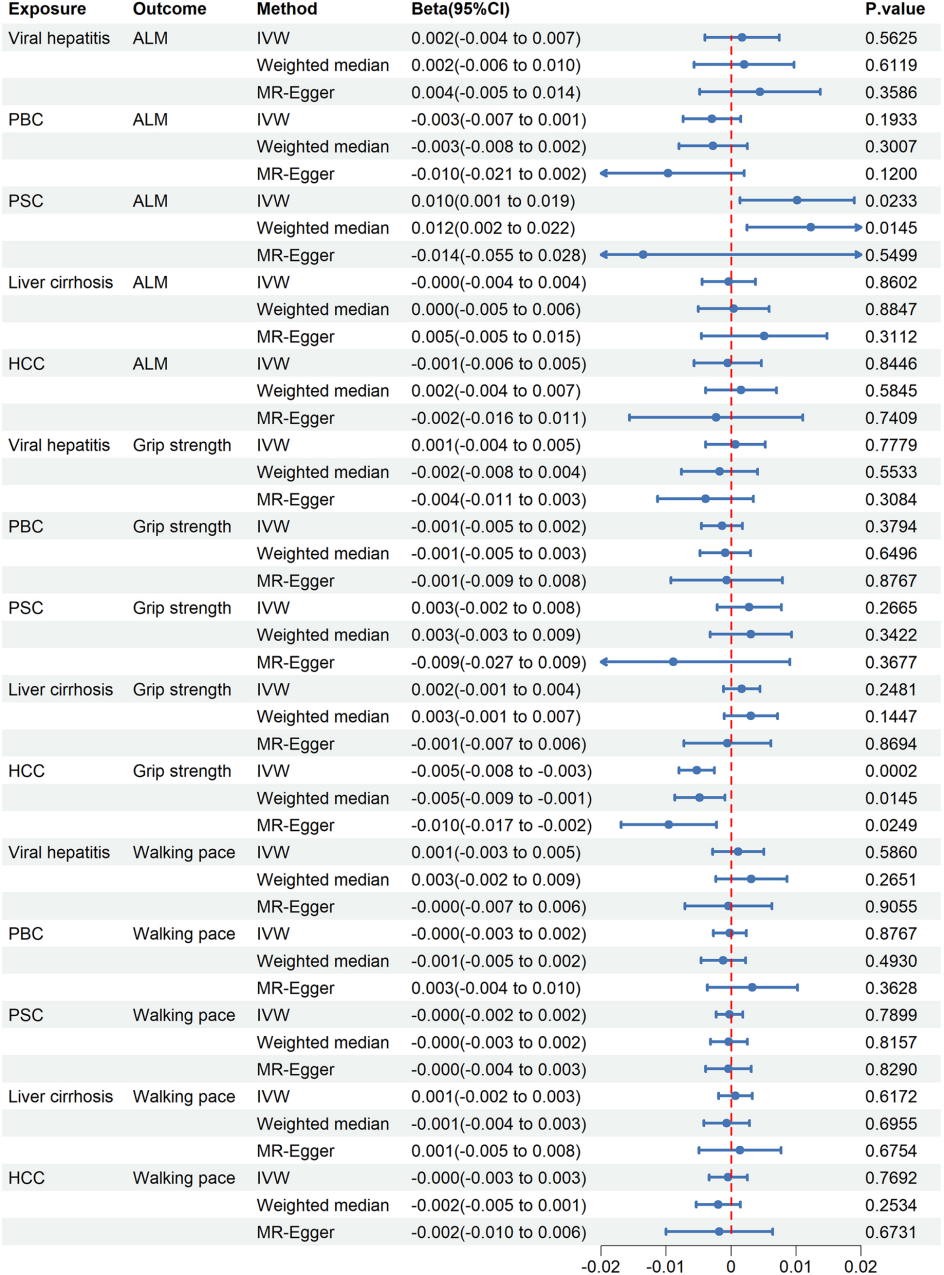
Causal effects of chronic liver diseases on sarcopenia. CI: confidence intervals; ALM: appendicular lean mass; IVW: inverse-variance weighted; PBC: primary biliary cholangitis; PSC: primary sclerosing cholangitis; HCC: hepatocellular carcinoma

Regarding muscle mass, the results from IVW (beta = 0.0102, 95% CI 0.0014–0.0190; *P* = 0.0233) and weighted median (beta = 0.0123, 95% CI 0.0024–0.0221; *P* = 0.0145) suggest a suggestive positive causal effect of PSC on ALM (Fig. 6). However, the MR-Egger analysis yielded inconsistent results (beta = − 0.0135, 95% CI − 0.0555 to 0.0284; *P* = 0.5499) (Fig. 6). Meanwhile, all three MR methods indicated that viral hepatitis, PBC, liver cirrhosis, and HCC had no causal effects on ALM (*P* > 0.05) (Fig. 6). In terms of muscle function, all three MR methods indicated that HCC led to a decrease in grip strength: IVW (beta = − 0.0053, 95% CI − 0.008 to − 0.0025; *P* = 0.0002), weighted median (beta = − 0.0048, 95% CI − 0.0087 to − 0.0010; *P* = 0.0145), and MR-Egger (beta = − 0.0096, 95% CI − 0.0169 to − 0.0023; *P* = 0.0249) (Fig. 6). Importantly, the CAUSE method further confirmed the negative causal effect of HCC on grip strength (ΔELPD = − 182.623, gamma = − 0.02, *P* = 5.5 × 10^–17^) (Additional file 1: Table S13). However, there was no evidence to suggest a causal impact of viral hepatitis, PBC, PSC, and liver cirrhosis on grip strength (*P* > 0.05) (Fig. 6). Moreover, no causal association was identified between the five chronic liver diseases and walking pace (*P* > 0.05) (Fig. 6).

### Causal effects of musculoskeletal disorders on chronic liver diseases

The reverse MR analysis evaluated the causal impact of 13 musculoskeletal disorder-related phenotypes on five chronic liver diseases. The comprehensive information regarding the causal effects of osteoporosis, osteoarthritis, and sarcopenia on chronic liver diseases is presented in Additional file 1: Tables S14–16.

Regarding the causal effects of osteoporosis on chronic liver diseases, the weighted median and MR-Egger methods respectively suggested a positive causal impact of LS-BMD on viral hepatitis (OR = 1.520, 95% CI 1.007–2.295; *P* = 0.0463) and eBMD on PBC (OR = 1.382, 95% CI 1.057–1.806; *P* = 0.0192) **(**Additional file 1: Table S14). However, the IVW method did not indicate a similar causal link **(**Additional file 1: Table S14).

Regarding the causal effects of osteoarthritis on chronic liver diseases, the IVW analysis suggested that hand osteoarthritis might increase the risk of liver cirrhosis (OR = 1.675, 95% CI 1.110–2.529; *P* = 0.0140). However, the MR-Egger method yielded inconsistent results (OR = 0.398, 95% CI 0.025–6.233; *P* = 0.5408) **(**Additional file 1: Table S15).

Regarding the causal effects of sarcopenia on chronic liver diseases, there is no evidence for the causal impacts of ALM and grip strength on chronic liver diseases (*P* > 0.05) (Additional file 1**: **Table S16). Additionally, the three MR analysis methods yielded inconsistent results about the causal effects of walking pace on liver cirrhosis (Additional file 1: Table S16).

### Sensitivity analysis

Table 2 presents the results of sensitivity analyses for the significant causal relationships between chronic liver diseases and musculoskeletal disorders. The *P* values of Cochran’s Q test for the associations between PSC and FA-BMD, PSC and overall osteoarthritis, as well as HCC and grip strength, were all greater than 0.05, indicating the absence of heterogeneity (Table 2). Importantly, given that the *P* values of the MR-Egger intercepts for the three exposures with the respective outcomes were all greater than 0.05, bias due to horizontal pleiotropy was ruled out (Table 2). Meanwhile, the MR-PRESSO global test also indicated that results of MR analyses were not affected by horizontal pleiotropy (*P* > 0.05), and the MR-PRESSO causal analysis further confirmed the significant causal relationships (*P* < 0.05) (Table 3). The complete results of heterogeneity and pleiotropy test for the forward and reverse MR analyses are available in Additional file 1: Tables S17 and Table S18. Furthermore, scatter plots (Additional file 2: Fig: S1) and symmetrical funnel plots (Additional file 2: Fig. S2) provided additional evidence against the presence of potential outliers. In addition, leave-one-out analyses indicated that the significant causal relationships were not driven by any single SNP (Additional file 2: Fig. S3).

**Table 2:** Heterogeneity and pleiotropy test of Mendelian randomization studies

Exposure	Outcome	Cochran’s Q test		MR-Egger	
		IVW (*P* value)	MR-Egger (*P* value)	Intercept	*P* value
PSC	FA-BMD	0.086	0.067	0.0081	0.633
PSC	ALL OA	0.183	0.158	− 0.0024	0.547
HCC	Grip strength	0.755	0.808	0.0021	0.239

IVW: inverse-variance weighted; PSC: primary sclerosing cholangitis; FA-BMD: forearm bone mineral density; ALL OA: any site osteoarthritis; HCC: hepatocellular carcinoma

**Table 3:** Results of MR-PRESSO analysis

Exposure	Outcome	Causal Estimate	SD	*P* value	Global Test (*P* value)
PSC	FA-BMD	− 0.045	0.018	0.023	0.129
PSC	ALL OA	0.012	0.005	0.040	0.175
HCC	Grip strength	− 0.005	0.001	0.001	0.777

MR-PRESSO: MR Pleiotropy RESidual Sum and Outlier; SD: standard deviation; PSC: primary sclerosing cholangitis; FA-BMD: forearm bone mineral density; ALL OA: any site osteoarthritis; HCC: hepatocellular carcinoma

In addition, the results of LCV model revealed the negative genetic correlation between PSC and FA-BMD (Rg = − 0.25, *P* = 2.17 × 10^–4^), the positive genetic correlation between PSC and overall osteoarthritis (Rg = 0.18, *P* = 1.56 × 10^–3^), and the negative genetic correlation between HCC and grip strength (Rg = − 0.14, *P* = 3.11 × 10^–4^) (Additional file 1: Table S19). Furthermore, the results of LCV model confirmed the partial causality among the three phenotypes mentioned above (0 < GCP < 1, *P* < 0.05) (Additional file 1: Table S19).

## Discussion

To the best of our knowledge, this study stands as the most comprehensive MR research to date assessing the causal relationships between chronic liver diseases and musculoskeletal disorders. This study found that PSC had suggestive causal effects on osteoporosis and osteoarthritis, and HCC exhibited a strong causal impact on sarcopenia. Specifically, PSC was associated with lower FA-BMD and an increased risk of overall osteoarthritis, while HCC was linked to decreased grip strength. However, the reverse MR analysis did not find any evidence of a causal effect of musculoskeletal disorders on chronic liver diseases. Complications arising from chronic liver diseases significantly impact patients’ physiological function and daily lives. This study underscores the importance of enhanced comprehensive management for patients with PSC and HCC to reduce the risk of musculoskeletal disorders and improve their long-term quality of life.

Musculoskeletal disorders related to chronic liver diseases are garnering increasing attention, and numerous observational studies have explored the risk of these disorders in patients with chronic liver diseases. The results of this study further confirm or complement the findings of some observational studies. An observational study involving 237 PSC patients found a higher prevalence of osteoporosis among these individuals, with a rate 23.8 times higher than the expected rate in the matched population [9]. Our study further demonstrated that the relationship between PSC and osteoporosis was site-specific, manifested as PSC reducing the BMD of forearm. Observational studies on the prevalence of osteoarthritis in patients with chronic liver diseases are relatively scarce. Our study, along with a nationwide cohort study in Denmark, suggests that liver cirrhosis does not affect the risk of osteoarthritis [14]. Additionally, this study is the first to identify a positive causal effect of PSC on overall osteoarthritis, filling the gap left by related observational studies. Furthermore, two retrospective studies involving 92 and 116 HCC patients respectively suggest that HCC patients tend to experience a decrease in lumbar skeletal muscle index and a higher prevalence of sarcopenia [48, 49]. While our study did not find a significant causal association between HCC and ALM, potentially due to differences in the specific muscle location considered, it uncovered a negative association between HCC and grip strength, addressing the research gap regarding the impact of HCC on muscle function in previous observational studies.

This study underscores a notable disparity between observational evidence and causal evidence regarding the relationship between chronic liver diseases and musculoskeletal disorders. Observational studies have indicated that viral hepatitis, PBC, and liver cirrhosis can significantly increase the risk of osteoporosis [12, 50, 51] and sarcopenia [12, 52, 53]. However, this MR study did not find a causal relationship between these three chronic liver diseases and osteoporosis or sarcopenia. It is important to recognize that conventional observational studies can only reveal the associations between chronic liver diseases and musculoskeletal disorders but cannot establish causality. The significant results in the aforementioned observational studies may be influenced by various factors. Firstly, observational studies inherently encounter challenges related to confounding factors. Variables like physical activity, dietary intake, and the use of specific medications related to chronic liver diseases can potentially confound the results of the observational studies [54, 55]. Secondly, observational studies cannot exclude the influence of reverse causality. For instance, although this MR study did not confirm a causal effect of liver cirrhosis on sarcopenia, another MR study found that sarcopenia could increase the risk of liver cirrhosis [56], which may partially explain the higher prevalence of sarcopenia observed in liver cirrhosis patients in observational studies. The disparities between the results of this MR study and previous observational research emphasize the necessity for an in-depth exploration of the biological mechanisms underlying the musculoskeletal disorders associated with chronic liver diseases, which can contribute to strengthening or clarifying the current understanding of the relationship between these two conditions.

The pathophysiological mechanisms behind musculoskeletal disorders stemming from chronic liver diseases are a cutting-edge research direction. Metabolic and secretory irregularities triggered by chronic liver diseases can precipitate bone loss. This chronic liver diseases-related metabolic bone disease characterized by reduced bone mass is known as hepatic osteodystrophy [57]. It is known that the liver plays a pivotal role in the activation of vitamin D [58], and vitamin D regulates bone mineralization and absorption to maintain BMD [59]. However, patients with PSC are prone to severe vitamin D deficiency [60, 61], suggesting that osteoporosis induced by PSC may be a type of hepatic osteodystrophy related to abnormal liver vitamin D metabolism. In addition, the liver is a central hub for lipid metabolism, and high blood lipid levels can induce the accumulation of lipids in cartilage, leading to osteoarthritis [62]. The reduction of cartilage matrix production and enhancement of cartilage matrix degradation induced by hypercholesterolemia play a crucial role in the occurrence of such metabolic osteoarthritis [63, 64]. Clinical studies have found that PSC patients typically have higher blood cholesterol levels [61, 65], pointing to excessive cholesterol accumulation as a potential mechanism for the causal effect of PSC on osteoarthritis. As for the causal relationship between HCC and sarcopenia, the underlying mechanisms may be multifactorial. Elevated release of cytokines like interleukin-1, interleukin-6, and tumor necrosis factor α, disrupted hormone levels including growth hormones and estrogen, and alterations in the tumor microenvironment in HCC patients may all impair skeletal muscle function [66].

It is noteworthy that in MR analyses of certain trait pairs, the IVW method did not show significant causal relationships, while the weighted median and MR-Egger approaches yielded significant results. This disparity primarily arises from differences in their algorithms and underlying assumptions. IVW assumes the validity of all IVs, uses the inverse of the variance as weights for fitting, and does not consider the presence of an intercept term in regression [39, 67]. MR-Egger assumes that most IVs are invalid, also utilizes inverse variance as weights for fitting, but incorporates an intercept term in regression [41, 68]. Weighted median assumes the validity of most IVs, and its results are derived from the median of the distribution function obtained by sorting the individual SNP effect values based on their weights [40]. It is essential to consider that each MR analysis method possesses its own strengths and limitations [69]. While IVW is commonly favored due to its robustness, significant findings from alternative methods like MR-Egger and weighted median should not be totally overlooked.

This study possesses several significant advantages. First and foremost, this research offered a more comprehensive and systematic evaluation of the association between chronic liver diseases and various musculoskeletal disorders compared to previous observational studies. Specifically, for osteoporosis and osteoarthritis, we focused not only on overall phenotypes but also on site-specific phenotypes; for sarcopenia, we focused on both muscle mass and muscle function. Second, this study minimized bias caused by confounding factors and reverse causality, thus presenting robust causal evidence. Importantly, the combined use of diverse MR analysis methods and multiple sensitivity analysis methods bolstered the reliability of the results. Additionally, musculoskeletal disorders are associated with various broadly defined cirrhosis [70, 71] and diverse types of cancer [72, 73]. To mitigate potential bias arising from these associations, the controls for liver cirrhosis and HCC in this study were defined to exclude broadly defined cirrhosis and all types of cancer, respectively, which ensured a higher degree of accuracy in the results of the causal analysis.

Nevertheless, several limitations should be acknowledged. Firstly, owing to the lack of relevant GWAS data, it was impossible to analyze the causal relationship between the severity of chronic liver diseases and musculoskeletal disorders. Secondly, although in numerous MR studies, including those involving chronic liver diseases, the use of FinnGen data alongside GWAS data from other Europeans has yielded meaningful results [74–77], underscoring the practicality and validity of FinnGen data for MR analyses, it is essential to acknowledge that the unique genetic composition of the Finnish population may introduce population-specific genetic variants and allele frequencies, distinguishing it from other European populations [25, 78]. As genetic variations serve as IVs, the distinct nature of the Finnish population’s genome may impact the validity of IVs and the generalizability of findings in MR analyses. Further substantial evidence is required to comprehensively evaluate the extent of this influence. Thirdly, the definition of cases for viral hepatitis in this research included all types of viral hepatitis, which prevented us from examining the causality between specific types of viral hepatitis and musculoskeletal disorders.

## Conclusions

In summary, this MR study comprehensively assessed the causal relationship between chronic liver diseases and musculoskeletal disorders, finding that PSC increases the risk of osteoporosis and osteoarthritis, while HCC can induce sarcopenia. These findings underscore the importance of enhancing comprehensive assessment and treatment for PSC and HCC patients to alleviate the burden of musculoskeletal disorders attributed to chronic liver diseases and improve their quality of life. Additionally, the disparities between the results of this MR study and previous observational studies highlight the need for in-depth investigations into the biological mechanisms underlying musculoskeletal disorders related to chronic liver diseases, in order to achieve a more precise understanding of their exact relationship from an etiological perspective.

### Supplementary Information

**Additional file 1: Table S1.** Definition of the cases and the controls in FinnGen. **Table S2.** Download link for GWAS data. **Table S3.** Information on instrumental variables for the causal effect of chronic liver diseases on musculoskeletal disorders. **Table S4.** Detailed information on instrumental variables for the causal effect of chronic liver diseases on osteoporosis. **Table S5.** Detailed information on instrumental variables for the causal effect of chronic liver diseases on osteoarthritis. **Table S6.** Detailed information on instrumental variables for the causal effect of chronic liver diseases on sarcopenia. **Table S7.** Information on instrumental variables for the causal effect of musculoskeletal disorders on chronic liver diseases. **Table S8.** Detailed information on instrumental variables for the causal effect of osteoporosis on chronic liver diseases. **Table S9.** Detailed information on instrumental variables for the causal effect of osteoarthritis on chronic liver diseases. **Table S10.** Detailed information on instrumental variables for the causal effect of sarcopenia on chronic liver diseases. **Table S11.** Steiger filtering results. **Table S12.** Excluded outlier SNPs. **Table S13.** Results of CAUSE method. **Table S14.** Mendelian randomization analysis of the causal effect of osteoporosis on chronic liver diseases. **Table S15.** Mendelian randomization analysis of the causal effect of osteoarthritis on chronic liver diseases. **Table S16.** Mendelian randomization analysis of the causal effect of sarcopenia on chronic liver diseases. **Table S17.** Heterogeneity and pleiotropy test of forward Mendelian randomization studies. **Table S18.** Heterogeneity and pleiotropy test of reverse Mendelian randomization studies. **Table S19.** Results of latent causal variable model.

**Additional file 2: Fig. S1.** Scatter plots.** (A)** Genetically predicted primary sclerosing cholangitis on forearm bone mineral density; **(B)** genetically predicted primary sclerosing cholangitis on any site osteoarthritis; **(C)** genetically predicted hepatocellular carcinoma on grip strength. **Fig. S2.** Funnel plots.** (A)** Genetically predicted primary sclerosing cholangitis on forearm bone mineral density; **(B)** genetically predicted primary sclerosing cholangitis on any site osteoarthritis; **(C)** genetically predicted hepatocellular carcinoma on grip strength. **Fig. S3.** Leave-one-out analyses.** (A)** Genetically predicted primary sclerosing cholangitis on forearm bone mineral density; **(B)** genetically predicted primary sclerosing cholangitis on any site osteoarthritis; **(C)** genetically predicted hepatocellular carcinoma on grip strength.

## Data Availability

The datasets supporting the conclusions of this study are available in FinnGen Study (https://www.finngen.fi/en), GWAS Catalog (https://www.ebi.ac.uk/gwas/), GEFOS (http://www.gefos.org/), GO Consortium (https://www.genetics-osteoarthritis.com), and IEU Open GWAS database (https://gwas.mrcieu.ac.uk/).

## Abbreviations

NAFLD: Nonalcoholic fatty liver disease
PBC: Primary biliary cholangitis
PSC: Primary sclerosing cholangitis
HCC: Hepatocellular carcinoma
RCT: Randomized controlled trial
GWAS: Genome-wide association study
MR: Mendelian randomization
IV: Instrumental variable
SNP: Single nucleotide polymorphism
BMD: Bone mineral density
TB-BMD: Total body BMD
FN-BMD: Femur neck BMD
LS-BMD: Lumbar spine BMD
FA-BMD: Forearm BMD
eBMD: Heel BMD
ALM: Appendicular lean mass
LD: Linkage disequilibrium
MR-PRESSO: MR Pleiotropy RESidual Sum and Outlier
IVW: Inverse-variance weighted
CAUSE: Causal Analysis Using the Summary Effect Estimates
ELPD: Expected log pointwise posterior density
LCV: Latent causal variable
GCP: Genetic causality proportion
OR: Odds ratio
CI: Confidence interval
PVE: Phenotypic variance explained
