# Supplementary material for: Genetic evidence of the causal relationship between chronic liver diseases and musculoskeletal disorders
Source: J Transl Med. 2024 Feb 6;22:138. doi: 10.1186/s12967-024-04941-1 (PMC10845502; doi:10.1186/s12967-024-04941-1)
Supplement: Supplementary file 2 — Additional file 2: Fig. S1. Scatter plots. (A) Genetically predicted primary sclerosing cholangitis on forearm bone mineral density; (B) genetically predicted primary sclerosing cholangitis on any site osteoarthritis; (C) genetically predicted hepatocellular carcinoma on grip strength. Fig. S2. Funnel plots. (A) Genetically predicted primary sclerosing cholangitis on forearm bone mineral density; (B) genetically predicted primary sclerosing cholangitis on any site osteoarthritis; (C) genetically predicted hepatocellular carcinoma on grip strength. Fig. S3. Leave-one-out analyses. (A) Genetically predicted primary sclerosing cholangitis on forearm bone mineral density; (B) genetically predicted primary sclerosing cholangitis on any site osteoarthritis; (C) genetically predicted hepatocellular carcinoma on grip strength. [file 12967_2024_4941_MOESM2_ESM.docx]

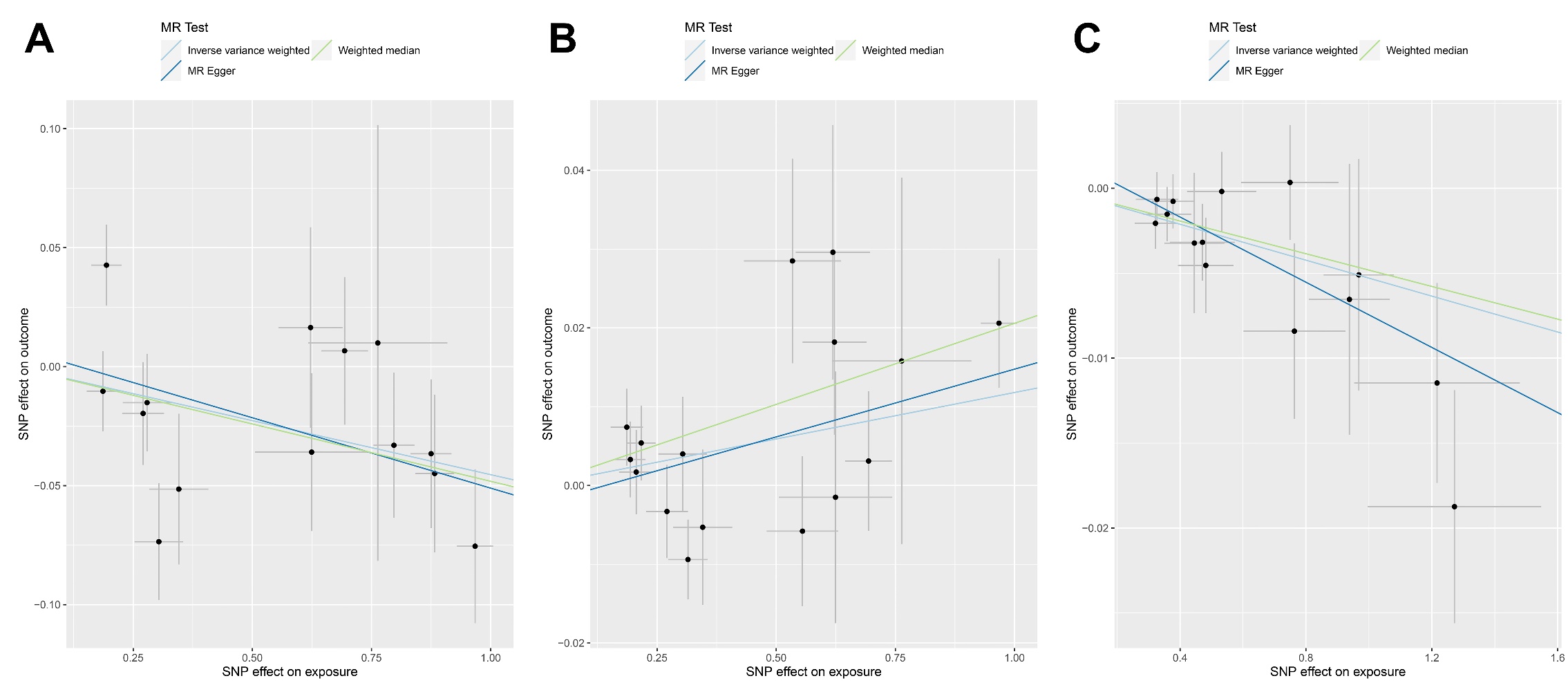


**Fig. S1 Scatter plots****. (A)** genetically predicted primary sclerosing cholangitis on forearm bone mineral density; **(B)** genetically predicted primary sclerosing cholangitis on any site osteoarthritis; **(C)** genetically predicted hepatocellular carcinoma on grip strength.


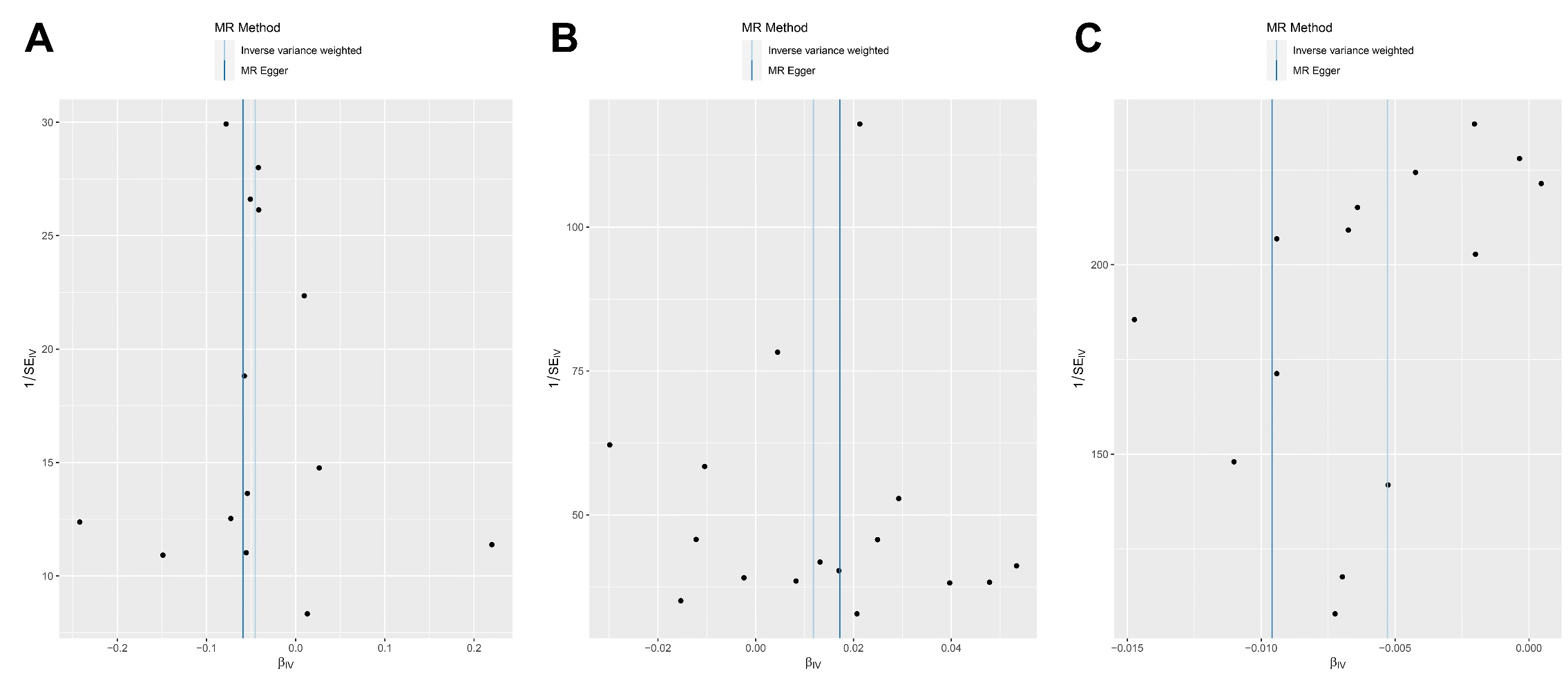


**Fig. S2 Funnel plots.** **(A)** genetically predicted primary sclerosing cholangitis on forearm bone mineral density; **(B)** genetically predicted primary sclerosing cholangitis on any site osteoarthritis; **(C)** genetically predicted hepatocellular carcinoma on grip strength.


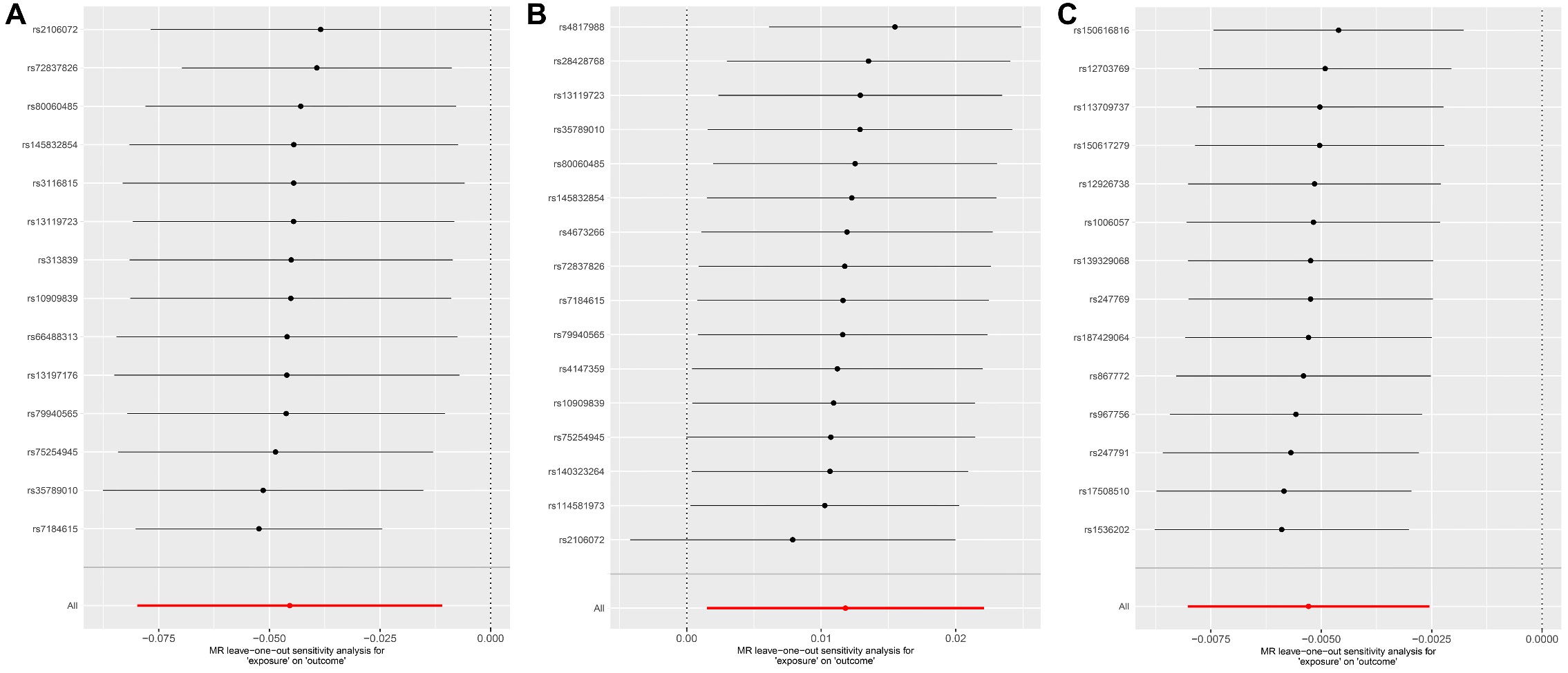


**Fig. S3 Leave-one-out analyses. (A)** genetically predicted primary sclerosing cholangitis on forearm bone mineral density; **(B)** genetically predicted primary sclerosing cholangitis on any site osteoarthritis; **(C)** genetically predicted hepatocellular carcinoma on grip strength.
